# Magnitude, Patterns, and Associated Predictors of Cardiovascular Events in Tetanus: A 2-Year, Single-Center, Ambidirectional Cohort Study Involving 572 Patients

**DOI:** 10.1093/ofid/ofad473

**Published:** 2023-09-20

**Authors:** Oanh Kieu Nguyet Pham, Bao Nhu Tran, Minh Cuong Duong, Thi Cam Nhung Do, Thi Lieu Pham, Minh Yen Lam, Louise Thwaites, Van Hao Nguyen

**Affiliations:** Faculty of Infectious Diseases, School of Medicine, Vietnam National University, Ho Chi Minh City, Vietnam; Viet Anh Department, Hospital for Tropical Diseases, Ho Chi Minh City, Vietnam; Faculty of Infectious Diseases, School of Medicine, Vietnam National University, Ho Chi Minh City, Vietnam; School of Population Health, University of New South Wales, Kensington, New South Wales, Australia; Viet Anh Department, Hospital for Tropical Diseases, Ho Chi Minh City, Vietnam; Emerging Infection Group, Oxford University Clinical Research Unit, Ho Chi Minh City, Vietnam; Emerging Infection Group, Oxford University Clinical Research Unit, Ho Chi Minh City, Vietnam; Emerging Infection Group, Oxford University Clinical Research Unit, Ho Chi Minh City, Vietnam; Department of Infectious Diseases, University of Medicine and Pharmacy at Ho Chi Minh City, Vietnam

**Keywords:** cardiovascular events, incidence, myocardial infarction, Takotsubo cardiomyopathy, tetanus

## Abstract

**Background:**

Cardiovascular events (CEs) remain the leading cause of death in patients with tetanus. We examined the incidence, patterns, and associated predictors of CEs among patients with tetanus in Vietnam.

**Methods:**

An ambidirectional cohort study was conducted on hospitalized adult patients with tetanus at the Hospital for Tropical Diseases between 2019 and 2020. Information on demographics, tetanus disease, CEs and outcomes were collected.

**Results:**

Among all 572 included patients, CEs accounted for 10.8% (95%CI 8.6–13.7%) and included Takotsubo cardiomyopathy (40.3%, 95%CI 29.0–52.8%), arrhythmia (19.4%, 95%CI 11.4–30.9%), sudden cardiac arrest (16.1%, 95%CI 9.0–27.2%), myocardial infarction (11.3%, 95%CI 5.6–21.5%), heart failure (6.5%, 95%CI 2.5–15.4%) and pulmonary embolism (6.5%, 95%CI 2.5–15.4%). CEs occurred from day 5 to 20 of illness. Among 62 CE patients, 21% (95%CI 12.7–32.6%) died and 61.3% (95%CI 48.9–72.4%) developed autonomic nervous system dysfunction (ANSD). Three-fourths (24/32) of patients with Takotsubo cardiomyopathy or myocardial infarction had ANSD. CEs were significantly associated with modified Ablett scores (AOR = 2.42, 95%CI 1.1–5.6, *P* = .04), underlying diseases (AOR = 2.7, 95%CI 1.1–6.8, *P* = .04) and overweight (AOR = 0.18, 95%CI .04–.8, *P* = .02).

**Conclusions:**

CEs are not rare and associated with high mortality. The most common CE is Takotsubo cardiomyopathy. CEs can occur at any stage of illness, with or without ANSD. To prevent mortality, it is pivotal to screen CEs in patients with tetanus, especially those with underlying diseases, high modified Ablett scores, and a normal or low BMI. More studies are needed to fully elucidate the impact of ANSD on the cardiovascular function and the CE associated mortality in tetanus.

## Teaser

Cardiovascular events (CEs) are not rare in tetanus and can occur at any stage of illness, regardless of autonomic nervous system dysfunction status. Ce screening should focus on patients with underlying diseases, high modified Ablett score, and normal/low body mass index.

Tetanus is caused by tetanus neurotoxin (tetanospasmin) produced by *Clostridium tetani* [1]. According to the Global Burden of Disease Study 2019, there were >73 000 tetanus cases and about 34 000 associated deaths worldwide [2]. In Vietnam, despite the extensive nationwide childhood immunization program, tetanus is not rare and remains a life-threatening disease [1]. There are approximately 250–350 cases admitted to the Hospital for Tropical Diseases (HTD)—the largest center for infectious diseases in southern Vietnam annually [3]. Most patients aged ≥16 years demonstrate an absence of primary series and booster tetanus vaccination in the community [4]. Recently, respiratory arrest, which used to be the most common cause of death from tetanus, has been well managed by tracheostomy procedure and ventilation [3]. Hence, cardiovascular system–related complications have been documented as a main cause of morbidity and mortality, although the etiology of cardiovascular events (CEs) in tetanus remains unclear [5]. A thorough understanding of the magnitude, manifestations, and outcomes of CEs in tetanus is needed to explore the etiology of these complications. This study examined the incidence, patterns, and associated risk factors of CEs, as well as treatment outcomes, in adult patients with tetanus in Vietnam.

## METHODS

### Study Design and Setting

A 2-year ambidirectional cohort study including a 1-year retrospective and 1-year prospective study was conducted at the HTD between January 2019 and December 2020 to examine the incidence, patterns, and associated predictors of CEs among tetanus patients. HTD receives patients with tetanus from across southern Vietnam [4]. The study was approved by the HTD's Ethics Committee (approval number 57/HĐĐĐ) and the Oxford Tropical Research Ethics Committee (approval number 517-20). Regarding the prospective study, informed consent was obtained from study participants or direct caregivers in case participants could not communicate. All patients aged ≥16 years with a clinical diagnosis of tetanus and without CEs at admission were invited to participate in the study. Tetanus management followed HTD's guidelines, including (1) preventing toxin uptake using antitoxin, antibiotic, and wound cleaning; (2) spasm control using benzodiazepines and muscle relaxant drugs; and (3) autonomic nervous system dysfunction (ANSD) control using magnesium sulfate and supportive care [3]. Patients are discharged if they have no laryngeal and respiratory muscle spasm, no longer require benzodiazepines or other sedatives, can perform self-care, and eat and drink normally [3].

A questionnaire was used to collect information on patients’ demographics, tetanus disease, CEs, and outcomes. Demographics included age, sex, residence address, overweight (body mass index [BMI] ≥25 kg/m^2^), underlying diseases (previous events of myocardial infarction [MI] or stroke, heart failure, hypertension, diabetes, chronic lung disease, arthropathy, liver disease, dementia, hemiplegia or paraplegia, chronic renal failure, gastric ulcer, cancer, AIDS, leukemia, or lymphoma), and tetanus vaccination history. Tetanus information comprised incubation period (number of days from having an apparent entry site to the development of the first symptom, which is mainly lockjaw), admission time (number of days from having the first symptom to admission), tetanus onset period (number of hours from having lockjaw to having the first larynx or muscle spasms), tetanus classification (generalized, local, and cephalic tetanus), ANSD (having at least 3 of 4 categories within 12 hours since the occurrence of disturbances of heart rate and blood pressure including heart rate >100 beats per minute; hypertension with systolic pressure >140 mm Hg; fluctuation of blood pressure with mean arterial pressure <60 mm Hg; and temperature >38°C without evidence of intercurrent infection) [6], ANSD onset period (number of days between when having the first tetanus symptom and when having the first symptom of ANSD), and severity of illness measured within the first day postadmission by using Sequential Organ Failure Assessment (SOFA) score, Dakar score, and Tetanus Severity Score (TSS), and the highest grade of modified Ablett score collected during the hospital stay [1]. The modified Ablett score is classified into 4 grades depending on the severity of illness including grade 1 (mild to moderate trismus but no spasm), grade 2 (moderate trismus and short spasm), grade 3 (severe spasm interfering with breathing), and grade 4 (grade 3 + ANSD) [1]. Ce information included types (Takotsubo cardiomyopathy [TCM], MI, arrhythmia, cardiac arrest, pulmonary embolism, and heart failure) and onset period (number of days between when having the first symptom of tetanus and when having the first symptom of Ce). In cases of TCM and MI, troponin I values at 0, 6, 12, 24, 48, 72, and 96 hours from the onset of CEs were collected. Echocardiography results and electrocardiography (ECG) abnormalities were also collected. Echocardiography results included the lowest ejection fraction (EF) value, images of apical ballooning, midventricular, basal, or focal left ventricular dyskinesia and myocardial contractility recovery period (number of days between the onset of CEs and the time when EF ≥50% and dyskinesias disappeared). ECG abnormalities included rapid sinus rhythm, ST elevation or depression, negative T wave, necrosis Q wave, deep and symmetrically T wave inversion, and prolonged QTc. TCM was identified based on the Heart Failure Association criteria [7]. Identifying MI was based on the European Society of Cardiology criteria (Supplementary Appendix 1) [8]. Ce diagnoses were made by the HTD's qualified intensivists, except TCM and MI, which were subsequently cross-checked by cardiologists. Echocardiography was performed by the HTD's experienced sonographers and intensivists. Angiography was not performed routinely because of its unavailability at the HTD, and external referrals of patients could not be made due to their severity of illness. Treatment outcomes included death and survival. Regarding the retrospective phase of the study, all patient data were collected based on medical records. Data collectors who were qualified medical doctors were consistently trained prior to the collection of data.

### Statistical Analysis

Data were analyzed using SPSS version 26 (IBM Corp, Armonk, New York). Categorical variables were presented as an absolute count and percentage. Regarding continuous variables, normally distributed data were presented as mean ± 1 standard deviation, while nonnormally distributed data were presented as median (interquartile range [IQR]). The χ^2^ test, χ^2^ test for trend, and Fisher exact tests were used to compare categorical data. Student *t* test and Mann-Whitney *U* test were used to compare continuous data with normal and nonnormal distribution, respectively. To identify predictors of CEs, a multivariable logistic regression model was developed and included all baseline variables including demographics (age, underlying diseases, previous MI, heart failure, hypertension, and overweight) and tetanus disease (tetanus onset period, admission time, Dakar score, TSS, and SOFA score measured within 24 hours postadmission as well as modified Ablett score and ANSD), which were statistically significant in the univariate analysis and other variables (previous ischemic stroke and diabetes) that were clinically judged to be associated with the current CEs. Alpha was set at 5% level.

## RESULTS

### Baseline Characteristics of 572 Study Participants

**Figure 1.**
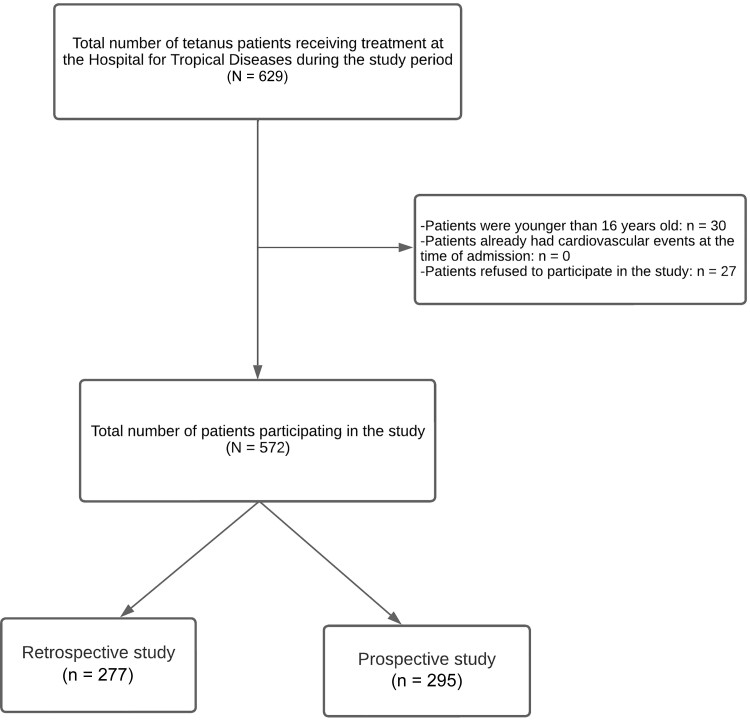
Flowchart of study participants.

There were 629 hospitalized patients with tetanus during the study period (Figure 1). Of these patients, 57 (9%) were excluded due to age restriction or refused to participate in the study. Hence, a total of 572 patients completed the study. Among these 572 participants, the mean age was 53.5 ± 15.7 years, most participants lived in rural areas, and most were male. Less than half of participants were overweight and had underlying diseases. Only 7 patients received tetanus vaccination or postexposure prophylaxis.

**Figure 2.**
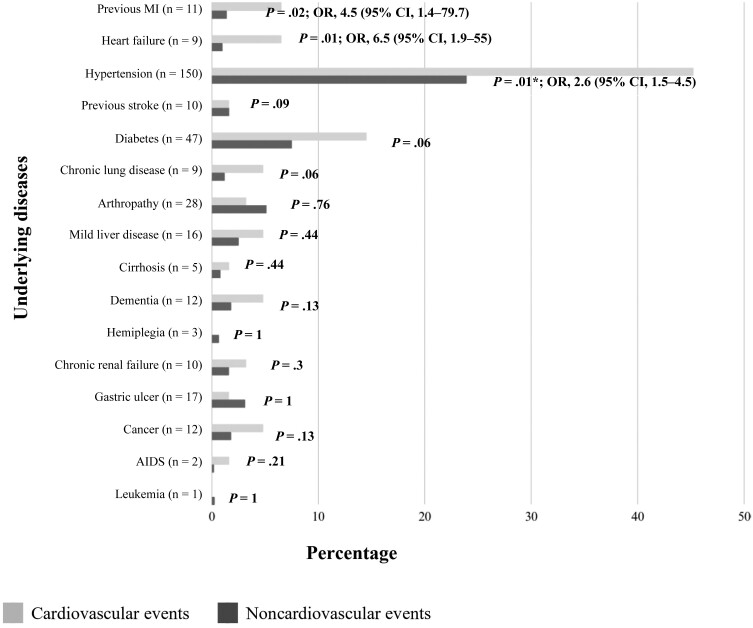
Distribution of underlying diseases by cardiovascular event status (n = 227). *χ^2^ test (all other *P* values from Fisher exact test). Abbreviations: CI, confidence interval; MI, myocardial infarction; OR, odds ratio.

The incidence of CEs was 10.8% (95% CI, 8.6%–13.7%). The mean age of the Ce group was statistically higher than that of those without Ce (non-Ce group) (Table 1). There was no statistical difference regarding sex distribution between the 2 groups. The proportion of overweight patients in the non-Ce group was statistically higher than that in the Ce group. In contrast, the proportions of patients with at least 1 underlying disease and those with previous events of MI, heart failure, and hypertension in the non-Ce group were statistically lower than those in the Ce group (Figure 2).

**Table 1.** Baseline Characteristics of Study Participants

Characteristics	Whole Population (N = 572)	Patients With Cardiovascular Event(s) (n = 62)	Patients Without Cardiovascular Event(s) (n = 510)	*P* Value	OR (95% CI)
Age, y, mean ± SD	53.5 ± 15.7	61.5 ± 15.8	52.6 ± 15.4	**<**.**01**^a^	…
Male sex	472 (82.5)	47 (75.8)	425 (83.3)	.14^b^	…
Rural residence	488 (85.3)	55 (88.7)	433 (84.9)	.42^b^	…
Overweight (BMI >25 kg/m^2^)	63 (11)	2 (3.2)	61 (12)	.**04**^b^	0.24 (.06–1)
Underlying diseases	227 (39.7)	42 (67.7)	185 (36.3)	**< .01** ^b^	3.7 (2.1–6.5)
Tetanus vaccination history^c^					
Tetanus vaccination	4 (0.7)	0	4 (0.8)	.56^d^	…
Postexposure prophylaxis	3 (0.5)	0	3 (0.6)	.45^d^	…

Data are presented as No. (%) unless otherwise indicated; Bold values: *P* < .05.

Abbreviations: BMI, body mass index; CI, confidence interval; OR, odds ratio; standard deviation.

a Student *t* test.

b χ^2^ test.

c One hundred five cases born after 1981; none of them received booster tetanus vaccination.

d Fisher exact test.

### Tetanus Disease Characteristics of Study Population

**Figure 3.**
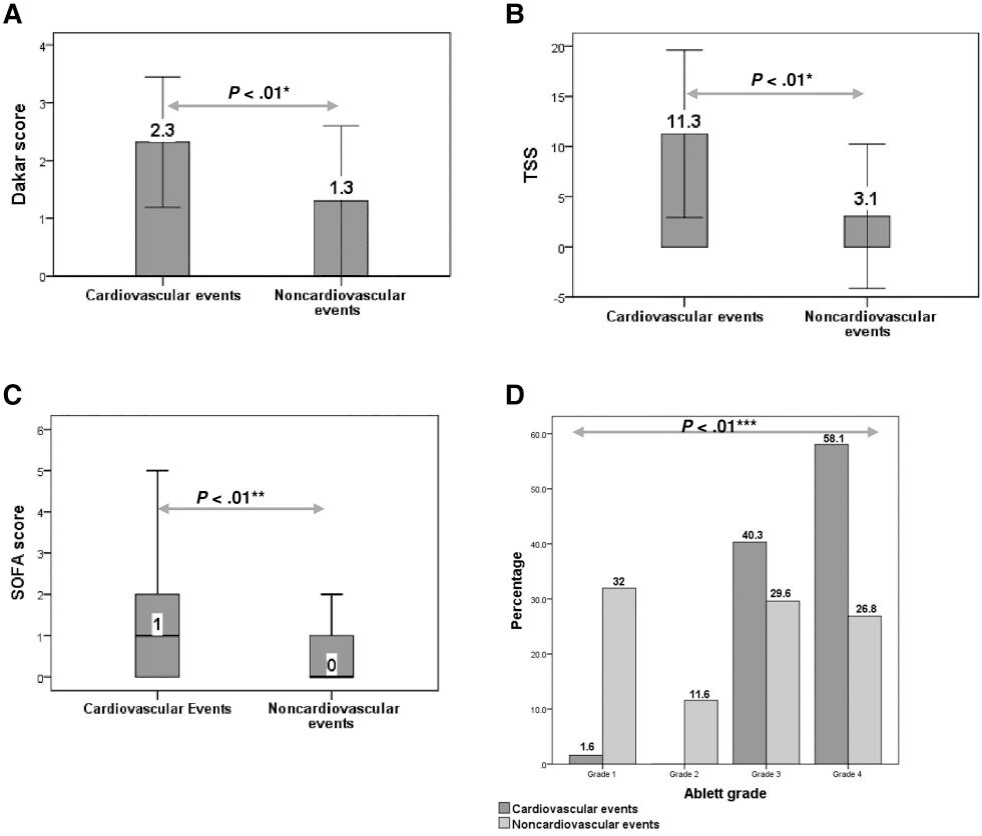
Distribution of Dakar score (A), Tetanus Severity Score (TSS) (B), and Sequential Organ Failure Assessment (SOFA) score (C) measured within 24 h postadmission as well as the highest Ablett grade (D) by cardiovascular event status (n = 572). *Student *t* test. **Mann-Whitney *U* test. ***χ^2^ test.

Among 572 study participants, >90% had lockjaw, difficulty in swallowing, and rigidity (Supplementary Appendix 2). The prevalence of laryngeal and muscular spasm was 17.1% and 42.3%, respectively. Approximately 5% of patients had fever on admission. The rates of dyspnea, rigidity, and muscular spasm in the Ce group were significantly higher than those in the non-Ce group. Approximately 60% of patients required mechanical ventilation, and most of them were those with CEs (98.4%) (Supplementary Appendix 2). The mean tetanus onset period (51.7 ± 43 vs 86 ± 55.7 hours, *P* < .01) and mean admission time (3.3 ± 2.3 vs 4.4 ± 2.7 days, *P* = .04) in the Ce group were statistically shorter than those in the non-Ce group (Table 2). The median SOFA score measured within 24 hours postadmission (1 [IQR, 0–2] vs 0 [IQR, 0–1], *P* < .01), mean Dakar score (2.3 ± 1.3 vs 1.3 ± 1.3, *P* < .01), and mean TSS (11.3 ± 8.3 vs 3.1 ± 7.2, *P* < .01) documented on the first day of admission in the Ce group were higher than those in the non-Ce group (Figure 3). Almost all (521/572 [91.1%]) patients in both groups developed generalized tetanus. There was an association between modified Ablett scores and CEs (*P* < .01) (Figure 3). The proportions of patients with ANSD (61.3% vs 26.9%, *P* < .01) and deaths (21% vs 2.4%, *P* < .01) in the Ce group were statistically higher than those in the non-Ce group.

**Table 2.** Characteristics of Tetanus Disease Among Study Participants

Characteristics	Whole Population (N = 572)	Patients With Cardiovascular Event(s) (n = 62)	Patients Without Cardiovascular Event(s) (n = 510)	*P* Value
Incubation period, d, median (IQR)	n = 433	n = 46	n = 387	.09^a^
	7 (5–12)	7 (5–8.3)	7 (5–13)	
Tetanus onset period, h, mean ± SD	n = 558	n = 62	n = 496	**<**.**01**^b^
	82.0 ± 55.4	51.7 ± 43.0	86.0 ± 55.7	
Admission time, d, mean ± SD	4.2 ± 2.6	3.3 ± 2.3	4.4 ± 2.7	.**04**^b^
Tetanus classification				.06^c^
Generalized tetanus	521 (91.1)	61 (98.4)	460 (90.2)	
Local tetanus	43 (7.5)	0 (0)	43 (8.4)	
Cephalic tetanus	8 (1.4)	1 (1.6)	7 (1.4)	
ANSD	175 (30.6)	38 (61.3)	137 (26.9)	**<**.**01^c^**
ANSD onset period, d, mean ± SD	n = 175	n = 38	n = 137	.10^b^
	6.6 ± 3.2	5.9 ± 2.9	6.8 ± 3.3	
Death	25 (4.4)	13 (21.0)	12 (2.4)	**<**.**01**^d^

Data are presented as No. (%) unless otherwise indicated; Bold values: *P* < .05.

Abbreviations: ANSD, autonomic nervous system dysfunction; IQR, interquartile range; SD, standard deviation.

a Mann-Whitney *U* test.

b Student *t* test.

c χ^2^ test.

d Fisher exact test.

### Patterns of Cardiovascular Events and Associated Characteristics Among 62 Study Participants

**Figure 4.**
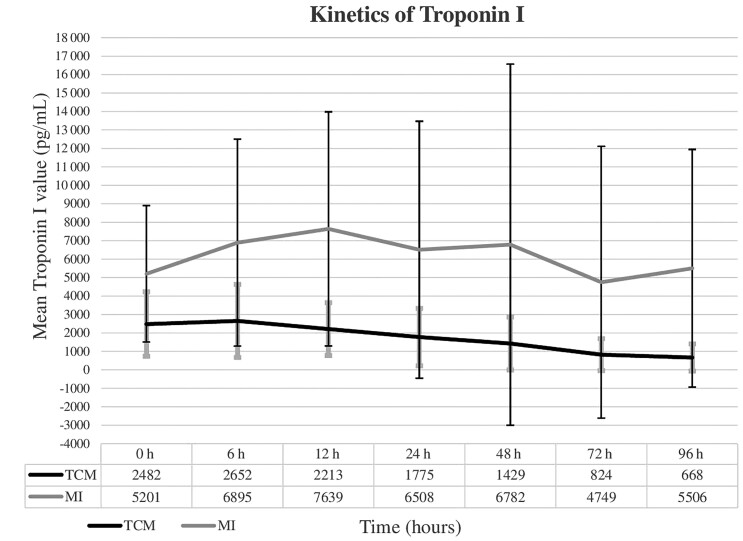
Kinetics of troponin I in patients with Takotsubo cardiomyopathy (TCM) and those with myocardial infarction (MI).

There were 62 patients with CEs including TCM (25/62, 40.3% [95% CI, 29.0%–52.8%]), arrhythmia (12/62, 19.4% [95% CI, 11.4%–30.9%]), sudden cardiac arrest (10/62, 16.1% [95% CI, 9.0%–27.2%]), MI (7/62, 11.3% [95% CI, 5.6%–21.5%]), heart failure (4/62, 6.5% [95% CI, 2.5%–15.4%]), and pulmonary embolism (4/62, 6.5% [95% CI, 2.5%–15.4%]) (Table 3). Among 12 patients with arrhythmia, the mean onset period was 16.5 ± 19.8 days, 6 did not develop ANSD, and 4 died. Among 10 patients with cardiac arrest, the mean onset period was 20.7 ± 14.4 days, 5 had ANSD, and 3 died. Among 25 patients with TCM, 19 developed ANSD and 2 died, whereas these values were, respectively, 6 and 4 in 12 patients with arrhythmia, 5 and 3 in 10 patients with cardiac arrest, 5 and 2 in 7 patients with MI, 1 and 1 in 4 patients with heart failure, and 2 and 1 in 4 patients with pulmonary embolism. The mean onset periods of Ce and ANSD were 6.1 ± 2.5 and 6.4 ± 2.7 days in the TCM group and 5.6 ± 5.2 and 3.8 ± 1.3 days in the MI group. Comparing patients with TCM and those with MI, the mean troponin I levels at the time of Ce onset and the mean peak troponin I levels in the TCM group were significantly lower than those in the MI group (2560 ± 1719 vs 5201 ± 3696 pg/mL, *P* = .01; and 3099 ± 2203 vs 8869 ± 6877 pg/mL, *P* < .01) (Table 4). The trend of the mean troponin I levels in TCM group increased slightly within the first 6 hours and then decreased daily (Figure 4). In contrast, it was biphasic with 2 peaks at 12 and 48 hours, followed by a slighter decrease in the MI group. In addition, there was no difference regarding the mean minimum EF value and characteristics of cardiac ultrasound and ECG between the 2 groups (Table 4). Among 25 patients with TCM, ECG abnormalities included rapid sinus rhythm (20/25), negative T wave (17/25), ST depression (8/25), ST elevation (7/25), and prolonged QTc (3/25). Among 4 cases with angiography, 3 cases with TCM had no blockage in coronary arteries, and 1 case with MI had a stent inserted (data not shown). Regarding echocardiography of 25 patients with TCM, mean lowest EF was 37.9% ± 15.8% and recovered after 1 week (7.1 ± 7 days); apical ballooning was documented in 9 (37.5%), midventricular dyskinesia in 10 (41.7%), and basal dyskinesia in 1 (4.2%) (Table 4). Among 7 MI cases, 4 had ST segment elevation, and 4 had negative T wave, of which 2 had necrosis Q wave and 1 had deep and symmetrical T wave inversion.

**Table 3.** Patterns and Characteristics of Cardiovascular Events, Autonomic Nervous System Dysfunction, and Mortality Among 62 Study Participants Developing Cardiovascular Events

Cardiovascular Events			Autonomic Nervous System Dysfunction		Death, no./No.
Types	Incidence, No. (%)	Onset Period, d, Mean ± SD	Frequency, no./No.	Onset Period, d, Mean ± SD	
Takotsubo cardiomyopathy	25 (40.3)	6.1 ± 2.5	19/25	6.4 ± 2.7	2/25
Arrhythmias^a^	12 (19.4)	16.5 ± 19.8	6/12	4.8 ± 2.3	4/12
Cardiac arrest	10 (16.1)	20.7 ± 14.4	5/10	6.4 ± 4.4	3/10
Myocardial infarction	7 (11.3)	5.6 ± 5.2	5/7	3.8 ± 1.3	2/7
Heart failure	4 (6.5)	9.0 ± 6.4	1/4	8	1/4
Pulmonary embolism	4 (6.5)	15.8 ± 19.5	2/4	6.5 ± 5	1/4

Abbreviation: SD, standard deviation.

a Arrhythmias included atrial fibrillation with rapid ventricular response (5/12), sinus bradycardia with ejection fraction <50% (2/12), irregular sinus rhythm (2/12), atrioventricular block third degree (1/12), Wolff-Parkinson-White syndrome (1/12), and paroxysmal supraventricular tachycardia (1/12).

**Table 4.** Comparison Between Patients With Takotsubo Myocardiopathy and Those With Myocardial Infarction Regarding Troponin I Levels and Cardiac Ultrasound and Electrocardiography Results

Characteristic	Variable	Takotsubo Myocardiopathy (n = 25)	Myocardial Infarction (n = 7)	*P* Value
Troponin I (pg/mL)	T0	2560 ± 1719	5201 ± 3696	.01^a^
		2490 (1130–39 996)	4523 (2426–9923)	
		24–6262	74–9993	
	Peak	3099 ± 2203	8869 ± 6877	<.01^a^
		2703 (1541–4747)	6107 (3778–13 020)	
		212–8640	820–21 162	
Cardiac ultrasound	Lowest ejection fraction, %	n = 24	n = 6	.96^a^
		37.9 ± 15.8	37.5 ± 11.8	
		36.6 (27.8–45.5)	41.5 (30.0–45.5)	
		12–69	15–47	
	Apical ballooning	9 (37.5)	2 (33.3)	1.00^b^
	Midventricular dyskinesia	10 (41.7)	1 (16.7)	.37^b^
	Basal dyskinesia	1 (4.2)	1 (16.7)	.37^b^
	Myocardial contractility recovery period, d	n = 17	n = 4	.42^a^
		7.1 ± 7.0	10.5 ± 8.6	
		5 (3–9)	9.5 (3–19)	
		1–30	1–22	
Electrocardiography	Rapid sinus rhythm	20 (80)	6 (85.7)	1.00^b^
	ST elevation	7 (28)	4 (57.1)	.19^b^
	ST depression	8 (32)	2 (28.6)	1.00^b^
	Negative T wave	17 (68)	4 (57.1)^c^	.67^b^
	Prolonged QTc	3 (12)	0	1.00^b^

Categorical variables are presented as No. (%), and continuous variables are presented as mean ± standard deviation, median (interquartile range), or min–max.

a Student *t* test.

b Fisher exact test.

c Two cases with necrosis Q wave and 1 case with deep and symmetrically T wave inversion.

### Predictors of Cardiovascular Events Among Study Participants

No other predictor for CEs was identified other than underlying disease (adjusted odds ratio [AOR], 2.79 [95% CI, 1.23–6.35]; *P* = .04), high grades of modified Ablett score (AOR, 2.37 [95% CI, 1.03–5.47]; *P* = .04), and overweight (AOR, 0.18 [95% CI, .04–.8]; *P* = .02) (Table 5).

## DISCUSSION

Our study found that CEs are not rare, especially in patients with severe tetanus. The manifestations of CEs vary and include TCM, arrhythmia, sudden cardiac arrest, MI, heart failure, and pulmonary embolism. CEs can develop at any stage of illness, even at a late stage, with or without the occurrence of ANSD. Both TCM and MI have nonspecific ECG changes, decreases in EF, and abnormalities of echocardiography. The kinetics of troponin I can help differentiate between TCM and MI. Patients with underlying diseases, high Ablett scores, or normal and low BMI have high risks of Ce development.

**Table 5.** Model for the Prediction of Cardiovascular Events

Characteristic	Adjusted OR (95% CI)
Age	1.01 (.98–1.04)
Underlying diseases	**2.79** (**1.23–6.35)**^a^
Previous myocardial infarction	0.31 (.66–1.49)
Heart failure	0.38 (.08–1.87)
Hypertension	1.02 (.26–2.26)
Previous ischemic stroke	4.55 (.46–44.45)
Diabetes	1.18 (.46–3.06)
Overweight	**0.18** (**.04–.80)**^b^
Onset time	0.99 (.98–1)
Admission time	0.94 (.79–1.12)
SOFA score measured within 24 h postadmission	0.98 (.77–1.26)
Dakar score measured within 24 h postadmission	0.95 (.69–1.29)
TSS measured within 24 h postadmission	1.05 (.99–1.11)
Modified Ablett score	**2.37** (**1.03–5.47)**^c^
Autonomic nervous system dysfunction	0.87 (.28–2.75)

Abbreviations: CI, confidence interval; OR, odds ratio; SOFA, Sequential Organ Failure Assessment; TSS, Tetanus Severity Score; Bold values: *P* < .05.

a *P*  = .04.

b *P*  = .02.

c *P*  = .04.

The considerably high mean age of patients and proportion of male sex in our study were similar to other studies [3, 9]. Given that Vietnam's Expanded Program on Immunization was introduced in 1981, people born before this year had not received tetanus vaccination [4]. This may explain the considerably old age of our patients. Among 572 patients with tetanus, only 7 patients received vaccination or postexposure prophylaxis, and none received full doses of vaccination. Vietnam's Maternal and Neonatal Tetanus Elimination program, introduced in 1991, has further increased the vaccination rate among women of reproductive age [4]. This could explain the high proportion of males among our study participants. Given that preventing tetanus infection by immunization is the primary prevention of tetanus disease and its complication including CEs, it is pivotal to increase booster coverage among adults and improve education of the community, especially men and the elderly, with a focus on the importance of tetanus vaccination.

The proportion of overweight patients in our study (11% [95% CI, 10.5%–11.6%]) was lower than that among adults in the general community in Vietnam (20.3% [95% CI, 15.2%–26.6%]) [10]. Most participants lived in rural areas where the prevalence of obesity is lower compared with the urban areas [11]. Our rate of laryngeal spasm was lower than that in Anuradha's study [12]. However, our rates of the remaining clinical symptoms including lockjaw, difficulty in swallowing, dyspnea, rigidity, muscular spasm, and fever were similar to those reported in other studies [12, 13]. The SOFA scores measured within 24 hours postadmission are considered as an effective tool to triage patients with sepsis into risk categories [14]. However, the role of day 1 SOFA scores in predicting the severity of tetanus has not been well understood. With a low median SOFA score of 0 (IQR, 0–1), most of our participants did not have any organ injury at admission, despite 39.7% of them having at least 1 underlying disease. There was no difference between Ce and non-Ce groups regarding day 1 SOFA score. The median SOFA scores in our study collected within 24 first hours after admission were lower than those reported in a study by Fan et al (5.3 ± 4.4) in which the timing of SOFA score identification was not provided and may have influenced the scores, provided that SOFA scores could change over time due to the disease progress [9]. Dakar scores and TSS have been commonly used to predict mortality in tetanus [15]. In our findings, there was no any association between these scores and CEs. Although CEs in patients with tetanus can lead to mortality, the frequency and patterns of these complications have not been well documented worldwide [16]. Our Ce rate is lower than the reported rate in Senegal (10.8% vs 20.9%) [17], which is probably due to different study contexts; our finding further demonstrates that CEs are not rare. The most commonly reported CEs include arrhythmias, blood pressure instability, MI, cardiac arrest, and cardiac ischemia [12, 16]. TCM has also been documented recently [18]. To the best of our knowledge, the first tetanus case with suspected TCM was reported in 1990 [19], followed by an official recognition of this health condition as a CE of tetanus in 2008 [18]. Despite this, information on the magnitude of TCM is scarce with a few cases reports worldwide [18, 19]. In Vietnam, although our study clinic is the leading center for tetanus [3], TCM was not documented previously because of the lack of bedside echocardiography in the intensive care unit (ICU). This is demonstrated by a study conducted at our study clinic in 2018 showing that among 14 of 164 (8.5% [95% CI, 5.2%–13.8%]) patients with tetanus with CEs, only MI (12/164) and arrhythmia (2/164) were documented [20]. The high MI frequency documented in this study probably included those with TCM but were misdiagnosed. Since late 2018 when ICU bedside echocardiography has been available, all CEs have been monitored and detected. In our study, the most common Ce was TCM (40.3%), followed by arrhythmia (19.4%), cardiac arrest (16.1%), MI (11.3%), heart failure (6.5%), and pulmonary embolism (6.5%).

TCM can mimic acute coronary syndrome but improves after several days to weeks or months [21]. In our study, due to the unavailability of coronary angiography at the study clinic and the severity of most of our patients, only 4 patients undertook angiography at other health center and included 1 case whose MI was confirmed. Among the remaining 3 cases without coronary artery occlusion, echocardiography showed acute heart failure with EF of 12%–53%, which completely recovered after 7–30 days. Other abnormalities in echocardiography included apical ballooning (2/3) and midventricular dyskinesia (2/3). Troponin I value was highest at Ce onset, decreased rapidly after 6 hours, and became normal after 28–42 days. All of these 3 patients recovered (data not shown). Similarly, the same changes in echocardiography and troponin I kinetics were also observed in other TCM patients who did not undertake angiography, all of which satisfied the Heart Failure Association criteria for TCM [7]. Regarding echocardiography, although the “octopus pot” image was not documented, other manifestations of left ventricular motion suggesting TCM were recorded and included reduced performance in the midventricular, apical, and basal regions. However, focal dyskinesia, a rare type of TCM, was not documented in our study [7]. The low EFs of our patients with TCM recovered after 1 week. Moreover, we found that among TCM patients, troponin I levels reached the peak after 6 hours and decreased rapidly, and the mean troponin I levels at the Ce onset were lower than those with MI. The peak of troponin I levels in TCM patients was also lower than that in patients with MI. The predisposing factor for TCM is emotional or physical stress and an excess catecholamine secretion [7, 18]. Regarding the latter, studies have found that the ANSD-mediated increases in the concentration of catecholamine in patients with tetanus could probably contribute to the development of TCM [22]. Although another recent study did not find an association between urinary catecholamine and heart rate, the study confirmed a possible association between catecholamine and ANSD [23]. This study measured urinary catecholamine only on day 5 after admission, which probably could not be the TCM onset time and the time catecholamine concentration reached its peak. Available published case reports of TCM in patients with tetanus documented that the TCM onset period was day 0–16 of illness, and all patients fully recovered [18, 19]. Most of our TCM patients developed ANSD. Our patients’ TCM developed at the end of the first week of illness, which was closed to the ANSD onset period, and 2 died. Considering these findings, we believe TCM can occur from the end of the first week of illness and may lead to mortality, although rare. Given the challenges in diagnosing TCM, physicians should pay attention to this Ce in patients with tetanus, especially those with ANSD.

The diagnosis of our 7 MI cases was based on cardiac specialists’ examination and real-time troponin I kinetics and ECG changes. The only case undertaking angiography was diagnosed with non-ST-elevation MI on day 17 of her disease with a sharply increased troponin I level (5690 pg/mL), new ST depression, and significant coronary artery obstruction. After coronary revascularization, her ECG improved quickly, while troponin I values became normal after >9 days (data not shown). The typical ECG changes suggesting MI were also documented in our patients with MI. Troponin I kinetics were seemly biphasic with 2 peaks that were typical for MI [24]. Like TCM, the mean MI onset period in our patients overlapped the period in which ANSD often occurs, and not all MI patients developed ANSD. Considering this, to prevent mortality, it is pivotal to detect acute MI in all patients with tetanus, regardless of ANSD status, especially during the first week of illness.

Cardiac arrhythmia has been well documented as a common Ce and a cause of death in tetanus, even in mild to moderate cases [16, 25]. In our study, arrhythmia was documented in 12 patients. Unlike TCM and MI in which the onset periods were close to the onset of ANSD, we noticed that arrhythmia developed considerably late and even in those without ANSD. Four patients with arrhythmia died. Our findings demonstrate that arrhythmia can occur at any stage of illness including a late stage (ie, after 2 week of illness), may not be associated with the occurrence of ANSD, and can lead to death.

Regarding sudden cardiac arrest, our rate was comparable to that of a report from Senegal (16.1% vs 24%) [17]. Like arrhythmia, we documented that cardiac arrest also appeared at a late stage of illness, in patients with and without ANSD, and could lead to mortality. ANSD is suspected as a main cause of unexpected cardiac arrest [26]. However, another study found that the levels of total catecholamines at the recovery stage were higher than those during the ANSD stage, and there were no significantly increased levels of catecholamines or tumor necrosis factor alpha, or evidence of cardiac systolic dysfunction in severe tetanus [27]. Given the limited knowledge of CEs in tetanus, we strongly believe that more robust studies are needed to explore the role of ANSD in CEs.

Deep venous thrombosis and pulmonary embolism can occur in tetanus due to long-term immobilization. Despite this, information on the burden of pulmonary embolism in tetanus is scarce [28]. In our study, there were 4 cases with pulmonary embolism, although all of them received long-term enoxaparin therapy to prevent deep venous thrombosis from the beginning of treatment. Considering this, periodic deep venous ultrasound evaluation should be performed to detect this Ce early. Unlike pulmonary embolism, cardiac failure is rarely reported in tetanus and could be due to the long-term use of β-adrenergic blocking agents in controlling autonomic dysfunction [29]. In our study, no patient was treated with β-blockers before CEs developed. We found that the onset of heart failure occurred at the beginning of the second week of illness when ANSD often developed [30]. Given the few studies about CEs in tetanus, the hemodynamic changes in tetanus are hypothesized to be complications of treatment [29]. Nevertheless, the true effects of tetanus on the cardiovascular system, especially in relation to the role of ANSD, remain unclear. As discussed previously, more studies would help in understanding the complexity of the pathogenesis of CEs in tetanus.

ANSD is not rare and may facilitate Ce development [16, 18]. However, our study did not find any association between ANSD and CEs, which concurs with another study in Brazil [16]. Although it remains unclear in patients with tetanus, risk factors for cardiovascular diseases in the general community are well documented and include hyperlipidemia, hypertension, diabetes mellitus, obesity, smoking, and sedentary lifestyle [31]. The patients’ smoking status, hyperlipidemia, and lifestyle were not documented in our study. We found that the prevalence of diabetes and hypertension in our study participants was comparable to those of the adult Vietnamese population [32]. However, we found no association between CEs and these single underlying diseases as well as other common cardiovascular risk factors including previous MI or stroke and heart failure. In contrast, patients with at least 1 underlying disease had higher risk of CEs, when all underlying diseases including diabetes and hypertension were grouped together. This can be attributable to a larger sample size of those with at least 1 underlying disease. In addition, patients with underlying diseases included those with 2 or more diseases that are cardiovascular risk factors, which may intensify the risk for CEs. In light of this, we believe future studies with larger sample sizes are needed to fully understand the association between common cardiovascular risk factors, such as diabetes and hypertension, and Ce development in tetanus. We found that overweight patients had a lower risk of CEs. Although we did not record the changes in patients’ weight during their hospital stay, patients with tetanus have a high risk of weight loss due to muscle wasting and digestive system impairment [33, 34]. Indeed, impaired function of the digestive system, which can lead to malnutrition, has been documented in patients with tetanus [33]. Patients with tetanus also experience muscle mass loss due to muscular contractions, excessive sweating, and sepsis [34]. Considering this, compared with overweight patients, those with an originally normal or low BMI could have a higher risk of developing undernutrition that may subsequently increase levels of stress and trigger cardiac disturbances. The modified Ablett score has been commonly used to assess the severity of tetanus [1]. Like another study, we found a positive association between the highest modified Ablett score during hospital stay and Ce development [35]. We also found a statistically significant association between CEs and death. Based on our findings, it is pivotal to be alert to Ce development in patients with tetanus with underlying diseases, high modified Ablett score, and normal or low BMI. More studies are needed to examine the impact of ANSD on cardiovascular function in tetanus as well as the Ce-associated mortality.

The study has some limitations. First, given that this was a single-center study, we may have missed patients who did not receive treatment at the HTD. Nevertheless, HTD is a leading tertiary hospital for infectious diseases and receives almost all patients with tetanus from southern Vietnam. Second, we were unable to perform angiographies for all suspected patients with TCM or MI. However, the diagnosis of TCM or MI in these patients was based on the clinical symptoms, kinetics of echocardiography, ECG, and troponin I, and cardiologists’ examinations. Third, given the nature of the 1-year retrospective study, we may miss some ECG results that were blurred over time. Fourth, the patients’ serial and highest SOFA scores were not recorded in this study, although this information would provide more insight into the progression of tetanus. Finally, despite our considerably long ambidirectional study conducted at a leading hospital for tetanus in Vietnam, our number of patients may not be sufficiently large to quantify all types of CEs.

In conclusion, CEs are not rare in severe patients with tetanus and can lead to death. The most common CEs including TCM, arrhythmia, sudden cardiac arrest, and MI can occur at any stage of illness, including a late stage, and may not be associated with the occurrence of ANSD. To prevent Ce-associated mortality in tetanus, it is pivotal to detect CEs in patients with tetanus, especially those with underlying diseases, high modified Ablett score, and a normal or low BMI. In low-resource settings where coronary angiography is not available, serial troponin I testing and cardiac ultrasound, in combination with close clinical monitoring of severe tetanus cases with Ablett score of 3–4, help in early detection of CEs. To prevent tetanus, increasing booster coverage among adults and improving community education with a focus on the importance of tetanus vaccination in adults are crucial. More studies are needed to have a full understanding of the impact of ANSD on cardiovascular function.

## Supplementary Material

ofad473_Supplementary_Data

## Supplementary Data

Supplementary materials are available at *Open Forum Infectious Diseases* online. Consisting of data provided by the authors to benefit the reader, the posted materials are not copyedited and are the sole responsibility of the authors, so questions or comments should be addressed to the corresponding author.

## Notes

***Author contributions.*** O. K. N. P., B. N. T., and M. C. D. conceived the study. All authors contributed to the protocol and design of the study. O. K. N. P., B. N. T., T. C. N. D., and T. L. P. contributed to project administration. O. K. N. P., B. N. T., T. C. N. D., T. L. P., M. Y. L., L. T., and V. H. N. contributed to the investigations performed in the study. O. K. N. P., B. N. T., and M. C. D. designed the statistical analysis and accessed and verified the underlying data. O. K. N. P., B. N. T., and M. C. D. drafted the report. All authors reviewed and approved the final report.

***Data sharing.*** De-identified data may be available upon reasonable request to the corresponding author.

***Patient consent.*** Regarding the prospective study, informed consent was obtained from study participants, or from direct caregivers in case participants could not communicate.

***Disclaimer.*** The funding sources had no involvement in the study.

***Financial support.*** This research was funded by the School of Medicine, Vietnam National University Ho Chi Minh City (grant number C2020-44-11 to B. N. T.) and the Oxford University Clinical Research Unit in Vietnam (grant number 07TS to O. K. N. P.).
