# Supplementary material for: Magnitude, Patterns, and Associated Predictors of Cardiovascular Events in Tetanus: A 2-Year, Single-Center, Ambidirectional Cohort Study Involving 572 Patients
Source: Open Forum Infect Dis. 2023 Sep 20;10(10):ofad473. doi: 10.1093/ofid/ofad473 (PMC10546955; doi:10.1093/ofid/ofad473)
Supplement: ofad473_Supplementary_Data [file ofad473_supplementary_data.zip › Appendix_1.docx]

**Appendix 1: Definitions of Takotsubo cardiomyopathy and myocardial infarction used in the study**

1. **Takotsubo Cardiomyopathy (TCM) definition:**

TCM was identified based on the following Heart Failure Association’s criteria.^1^

| Criterion | Characteristics |
| --- | --- |
| 1 | The left or right ventricular muscular abnormalities with transient regional wall motion which are regularly but not always, preceded by a stressful trigger |
| 2 | The abnormal regional wall movements extend beyond a single epicardial vascular distribution and cause the involved ventricular segments failure |
| 3 | No evidence of current acute atherosclerotic coronary artery disease such as acute coronary rupture, coronary dissection, and thrombus |
| 4 | The appearance of new and reversible abnormal ECG including ST-segment elevation, ST depression, left bundle branch block, T-wave inversion, or QTc prolongation during the acute phase (3 months) |
| 5 | The significant increase of natriuretic peptide or N-terminal pro-B-type natriuretic peptide during the acute phase |
| 6 | Imbalance between the increase of troponin I levels and the amount of dysfunctional heart muscle segments present |
| 7 | Rapid recovery of left ventricular EF within 3–6 months |

1. **Myocardial Infarction (MI) definition:**

MI was identified based on the following European Society of Cardiology’s criteria.^2^

| Criterion | Characteristics |
| --- | --- |
| 1 | Having acute coronary syndrome with or without significant ST segment elevation or new left bundle branch block |
| 2 | Development of pathological Q waves in ECG |
| 3 | Imaging evidence of new loss of viable myocardium or new regional wall motion abnormality |
| 4 | Having evidence of intracoronary thrombus by angiography or autopsy or sharp T, troponin I increase and having compatible changes between troponin I and ECG |

References:

1. Rawish E, Stiermaier T, Santoro F, Brunetti ND, Eitel I. Current Knowledge and Future Challenges in Takotsubo Syndrome: Part 1-Pathophysiology and Diagnosis. *Journal of clinical medicine.* 2021;10(3).

2. Kristian Thygesen JSA, Allan S. Jaffe, Maarten L. Simoons, Bernard R. Third universal definition of myocardial infarction. *European Heart Journal.* 2012;33:2551–2567.
