# Supplementary material for: Magnitude, Patterns, and Associated Predictors of Cardiovascular Events in Tetanus: A 2-Year, Single-Center, Ambidirectional Cohort Study Involving 572 Patients
Source: Open Forum Infect Dis. 2023 Sep 20;10(10):ofad473. doi: 10.1093/ofid/ofad473 (PMC10546955; doi:10.1093/ofid/ofad473)
Supplement: ofad473_Supplementary_Data [file ofad473_supplementary_data.zip › Appendix_2.docx]

**Appendix 2: Clinical characteristics of 572 study participants**

| **Clinical characteristics** | **Whole population**  **(n=572)** | **Patients with cardiovascular event(s)**  **(n=62)** | **Patients without cardiovascular event(s)**  **(n=510)** | **P** | **OR**  **(95%CI)** |
| --- | --- | --- | --- | --- | --- |
|  | **n%** | | |  |  |
| Lockjaw | 571 (99.8) | 62 (100) | 509 (99.8) | 0.72* |  |
| Difficult swallowing | 564 (98.6) | 62 (100) | 502 (98.4) | 1** |  |
| Laryngeal spasm | 98(17.1) | 15 (24.2) | 83 (16.3) | 0.11* |  |
| Dyspnea | 128 (22.4) | 24 (38.7) | 104 (20.4) | **0.01*** | **2.5 (1.4 – 4.3)** |
| Rigidity | 520 (90.9) | 61 (98.4) | 459 (90) | **0.03*** | **6.8 (0.9 – 49.9)** |
| Muscular spasm | 242 (42.3) | 34 (54.8) | 208 (40.8) | **0.03*** | **1.8 (1 – 3)** |
| Fever | 28 (4.9) | 4 (6.5) | 24 (4.7) | 0.53** |  |
| Ventilation | 348 (60.8) | 61 (98.4) | 287 (56.3) | **<0.01*** | **47.4**  **(6.5 – 344.6)** |

*Chi-squared test

**Fisher’s Exact test
